# A comprehensive Malabar Spinach dataset for diseases classification

**DOI:** 10.1016/j.dib.2025.111532

**Published:** 2025-04-06

**Authors:** Mushfiqur Rahman, Md Al Mamun

**Affiliations:** Department of Computer Science and Engineering, Daffodil International University, Dhaka, Bangladesh

**Keywords:** Malabar Spinach, Machine vision, Automatic detection system, Agriculture, Computer vision

## Abstract

This study focuses on the urgent need to increase detection of diseases in Malabar Spinach, a valuable leaf vegetable crop which is at risk from several disease types including Anthracous leaf spot and Straw mite infestation. There is still a lack of research focused on Malabar spinach, although advances in machine vision have considerably increased the detection of largescale crop diseases. By developing and evaluating machine vision algorithms specifically designed for accurate detection of diseases in Malabar spinach, this research aims to fill this gap. To achieve this, a comprehensive dataset comprising images of both healthy and diseased Malabar Spinach plants is utilized for training, testing, and validation purposes. This study seeks to develop reliable disease detection models through the examination of different image processing techniques and deep learning algorithms such as ResNet50. In particular, the performance of these models is rigorously evaluated on the basis of a set of standardized evaluation metrics which aim to achieve an overall test accuracy of 94%. The results of this research will have a major impact on the cultivation of Malabar spinach in terms of precision farming techniques and effective crop management practices. This study will contribute to the wider objectives of agricultural sustainability and food security, through increasing crop productivity and reducing yield losses. In the end, it is intended to strengthen the resilience of farming communities dependent on Malabar Spinach crops by providing farmers and experts with efficient tools for detecting diseases.

Specifications Table

**Table:** 

Subject	Computer Science
Specific subject area	Computer Vision, Image Processing, Leaf Vegetables, Deep Learning.
Data format	Raw images
Type of data	jpg
Data collection	A comprehensive dataset has been created to develop machine vision algorithms for detecting Malabar Spinach diseases. The dataset includes 603 original images representing three classes such as Anthracnose leaf spot, Straw mite, and Healthy Malabar Spinach. These images were collected from real fields with the assistance of experts from agricultural research institutes. Additionally, the dataset has been augmented with 5868 images generated from the original ones using some techniques like flipping, shearing, zooming, and rotation. This augmentation aims to improve the accuracy and robustness of the algorithms by providing a larger and more diverse set of data for training and testing.
Data source location	**Location:** Local Agriculture field. **Zone:** Birulia, Ashulia, Savar, Dhaka. **Country:** Bangladesh
Data accessibility	**Repository name:** Malabar Spinach dataset for diseases classification using deep learning approach. **Data identification number:**10.17632/n56pn9fncw.2 **Direct URL to data:**https://data.mendeley.com/datasets/n56pn9fncw/2

## 1 Value of the Data

• There is significant value in the data on Malabar spinach diseases across different agricultural and technology sectors. It is an essential tool for monitoring and maintaining the health of the Malabar Spinach crop, enabling farmers and agricultural experts to identify and address the disease as soon as possible [1]. Farmers can implement effective management strategies to reduce crop losses and increase yields through the identification of disease patterns and prevalence. In addition, the availability of complete data sets facilitates R&D efforts, about machine vision algorithms and automatic disease detection systems. This will contribute to the development of precision farming, while promoting healthy production practice for crops [2]. In addition, data is a key tool for training and education of agricultural professionals and researchers who are empowered with the knowledge and tools needed to address disease problems effectively. Finally, the economic impact of Malabar Spinach diseases data cannot be understated, as it enables stakeholders to make informed decisions to mitigate economic losses and sustain the profitability of Malabar Spinach cultivation [3]. Overall, the information on Malabar Spinach disease has been a cornerstone for improving crop health, optimizing management practices, and stimulating innovation to ensure economically viable production of Malabar spinach.
• Researchers from various fields can learn lots of things about the Malabar spinach disease from these data sets. First, they can be used as the basis for developing and refining machine learning algorithms and computer vision models adapted to disease detection and classification in Malabar spinach plants. To enhance the accuracy and reliability of disease detection systems, researchers will be able to study novel techniques and methodologies by exploiting these data. In addition, these data sets allow researchers to compare their approaches with existing models based on similar data and support developments in the field [4]. Furthermore, researchers can utilize the datasets for transfer learning, augmenting model performance by fine-tuning pre-trained models on the provided data. In addition, such datasets provide a basis for interdisciplinarity and bridge the gap between agriculture and technology fields to address some of the challenges faced by farmers. Overall, these data sets not only contribute to scientific research but also serve as educational resources that allow students and professionals to learn more about the intersection of agriculture and data science.

## 2 Background

Malabar spinach, a vital leaf vegetable grown in tropical and subtropical areas, is sought for its nutritional value as well as adaptability. However, it is vulnerable to diseases such as Anthracnose leaf spot and infestation by pests like the Straw mite which results in substantial crop losses if not dealt with immediately [5]. However, research in this area on Malabar Spinach has not been sufficiently developed despite the promise of machine vision and AI for detection of crop diseases. In addition, there are specific challenges in detecting diseases due to the special characteristics of Malabar Spinach such as leaf morphology and disease susceptibility. Therefore, this study is aimed at developing computer vision algorithms that are specifically designed for the detection of Malabar Spinach disease in a precise manner. This research aims to contribute to precision farming and sustainable crop management practices specific to the cultivation of Malabar spinach using a comprehensive database of images of healthy and diseased plants. The study aims at providing valuable information and tools for farmers and agricultural experts to effectively manage the Malabar Spinach disease, ultimately improving crop productivity and livelihoods in areas where they are grown, by addressing this gap in research.

## 3 Data Description

Malabar Spinach disease is a widespread problem affecting the productivity and quality of agricultural production. It has a detrimental impact on the quality of Malabar Spinach crops. Malabar Spinach is a leafy green vegetable frequently grown for its nutritional value and taste. However, under certain non-biological circumstances, diseases can severely harm the yield and quality of Malabar Spinach, resulting in significant economic losses for farmers [6]. Traditional methods of diagnosing these diseases are often time-consuming, labor-intensive, ineffective, and subjective.

In recent years, computer vision approaches have shown great promise in addressing the challenges of disease classification and detection in Malabar Spinach crops.

To develop machine vision-based algorithms for detecting Malabar Spinach diseases, a comprehensive dataset has been curated. This dataset comprises images representing various Malabar Spinach diseases, including Anthracnose_leaf_spot, Straw_mite, and Healthy Malabar Spinach. The classification of Malabar Spinach diseases was accomplished with the collaboration of experts from agricultural research institutes [7]. For this study, we have published the Malabar Spinach dataset in two folders: one containing the original dataset and the other containing the augmented dataset. Each folder is organized into three subfolders: Anthracnose, Straw, and Healthy [8].

In total, 603 photos of Malabar Spinach leaves have been captured from fields. Furthermore, 5868 augmented images were created from the originals to expand the dataset using techniques such as flipping, shearing, zooming, and rotating. This augmentation is crucial for enhancing the accuracy and robustness of the machine vision algorithms for detecting and classifying Malabar Spinach diseases. The main challenge during data collection was obtaining photos with noisy backgrounds and inconsistent lighting, complicating object identification and segmentation.

The potential applications include automated disease diagnosis using machine learning models, real-time crop health monitoring in precision agriculture, facilitating plant pathology research, developing training tools, and optimizing commercial agriculture practices to reduce disease-related losses and ensure produce quality.

## 4 Experimental Design, Materials and Methods

### 4.1 Materials for raw data collection

First, we collect raw data from different agriculture fields by using different smart phones. The smart phones are Samsung J4+, Redmi Note 8, and POCO X2. There are special configurations for every smart phone. Samsung j4+ has 13MP real camara but Redmi Note-8 and POCO X2 have different lens types with quad -lens reflex digital camara. Also, all devices are equipped with LED flash and HDR functions.

### 4.2 Data annotation

The dataset has been manually annotated. This process involved experts from agricultural department carefully labeling each image to ensure accurate identification and classification of diseases.

### 4.3 Data augmentation

Data augmentation is a method of increasing the diversity and size of data sets by applying various transformations to their original samples. In the context of developing machine vision algorithms for detecting Malabar Spinach diseases, data augmentation plays a crucial role in enhancing the model's accuracy and robustness by exposing it to a wider range of variations and scenarios that may occur in real-world conditions. Here's we applied for our data augmentation:

• Rotation
• Zoom
• Flip left and Right
• Flip top and bottom

For rotation, we give parameters with the value of probability 70 %, max left rotation and max right rotation is 10. In zooming part, probability 50 %, minimum factor is 1.1 and maximum factor is 1.5. After zooming we reset rotation value with different number and then flip left to right with probability 50 %. After flipping set random zooming with probability 50 % and percentage area is 80 %. And finally apply flip top to bottom with probability 50 % (Table 1).

**Table 1:** Sample of three class data.

Name of the class	Description	Sample data
Healthy	In this class data, generally full fresh and has a look of natural with proper color of green. Also, there is no spot.	
Anthracnose leaf spot	Usually, these data show round, lopsided spots on the leaves. Anthracnose is a fungal disease. It can spread very quickly in warm (80 degrees F), wet weather, especially if air circulation is poor [9]. At first, these spots seem wet or dark brown, but later they become dead.	
Straw mite	These images typically show symptoms associated with mite infestation, such as small yellow or brown spots curled on the leaves, stippling or bronzing of the leaf's surface and distortion. Its basic symptoms are holes in leaves and deformed leaves [10].	

**Fig. 1:**
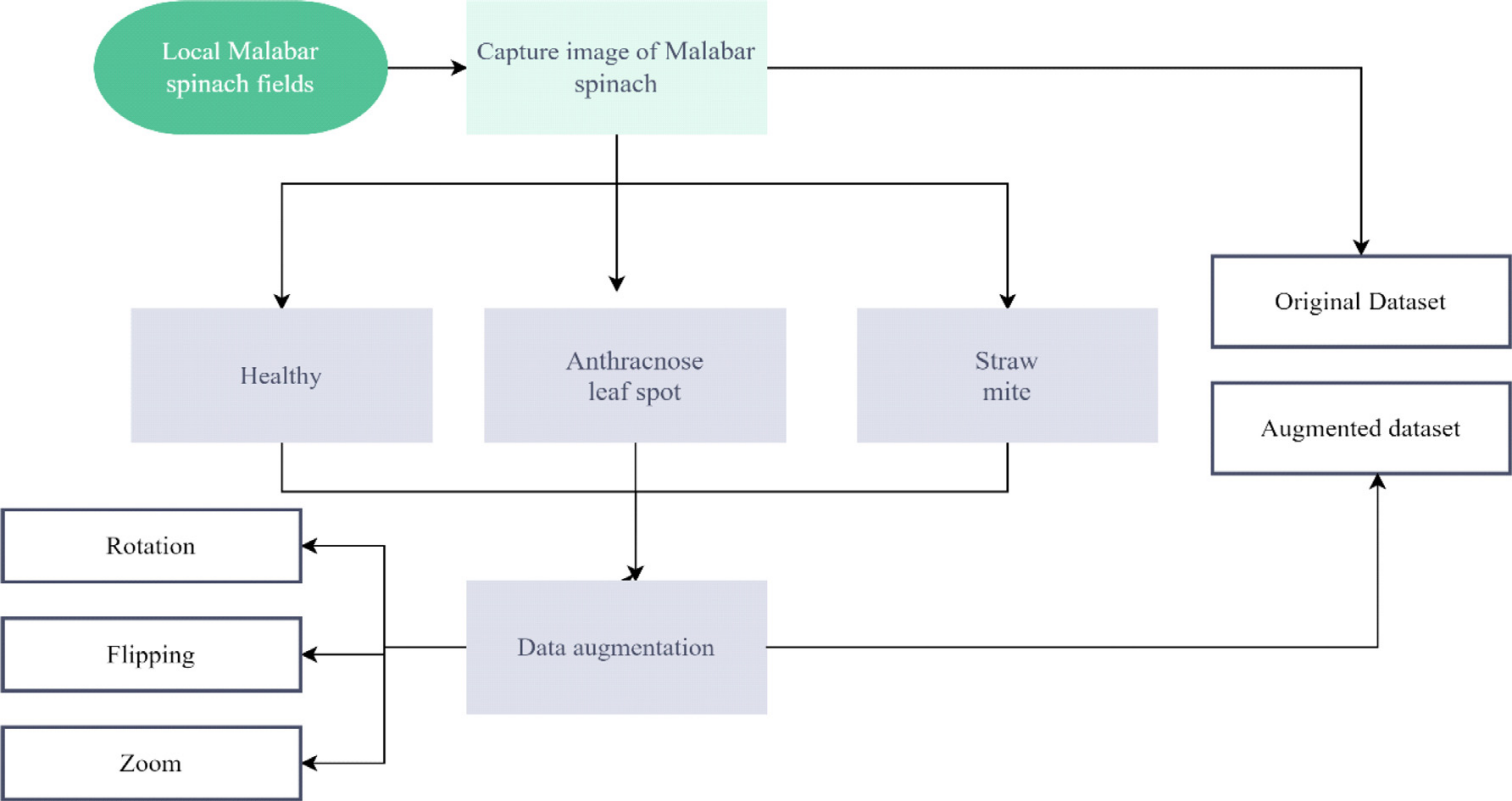
Malabar spinach dataset generation process.

In the above Fig. 1, we can find a clear idea of the data collection process. First, we captured images of Malabar spinach from local agriculture fields and stored them in their respective classes. That is our original dataset. After that, we applied rotation, flipping and zooming for data augmentation. And then stored as augmented data with their respective classes (Table 2).

**Table 2:** Augmented images of Malabar spinach dataset.

In Table 3, represent Malabar spinach dataset statistics based on original data and augmented data. In the original dataset, we have 150 healthy data, after applying augmented technic healthy data becomes available 1500. That similar process makes Anthracnose leaf spot data and Straw mite data becomes available respectively 2014 and 2228.

**Table 3:** Malabar spinach dataset statistics.

Name of the class of dataset	Number of images in original dataset	Number of images of augmented dataset
Healthy	150	1500
Anthracnose leaf spot	214	2014
Straw mite	239	2228

### 4.4 Classification methodology

**Fig. 2:**
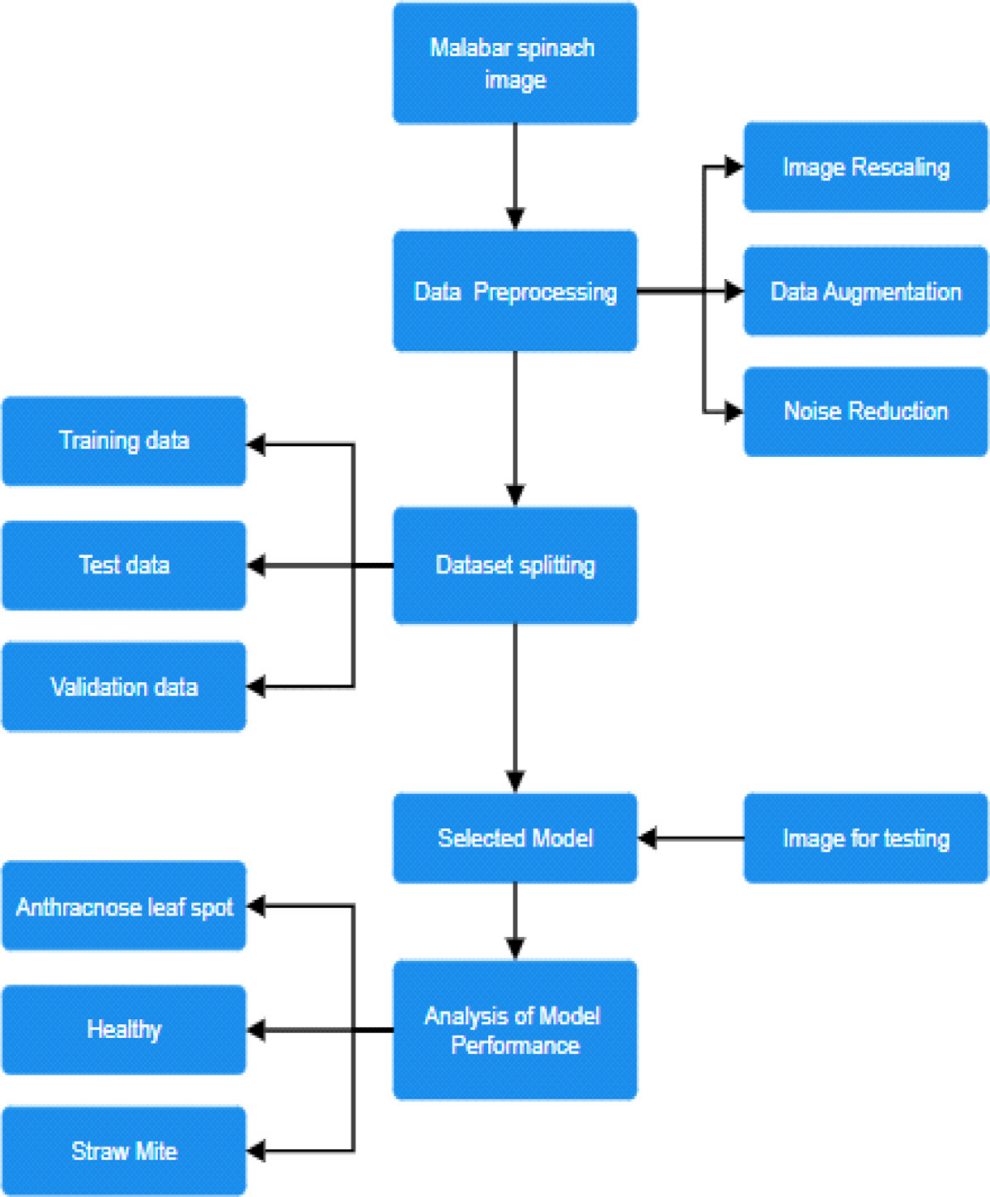
Procedure of work for classifying Malabar spinach diseases.

**Fig. 3:**
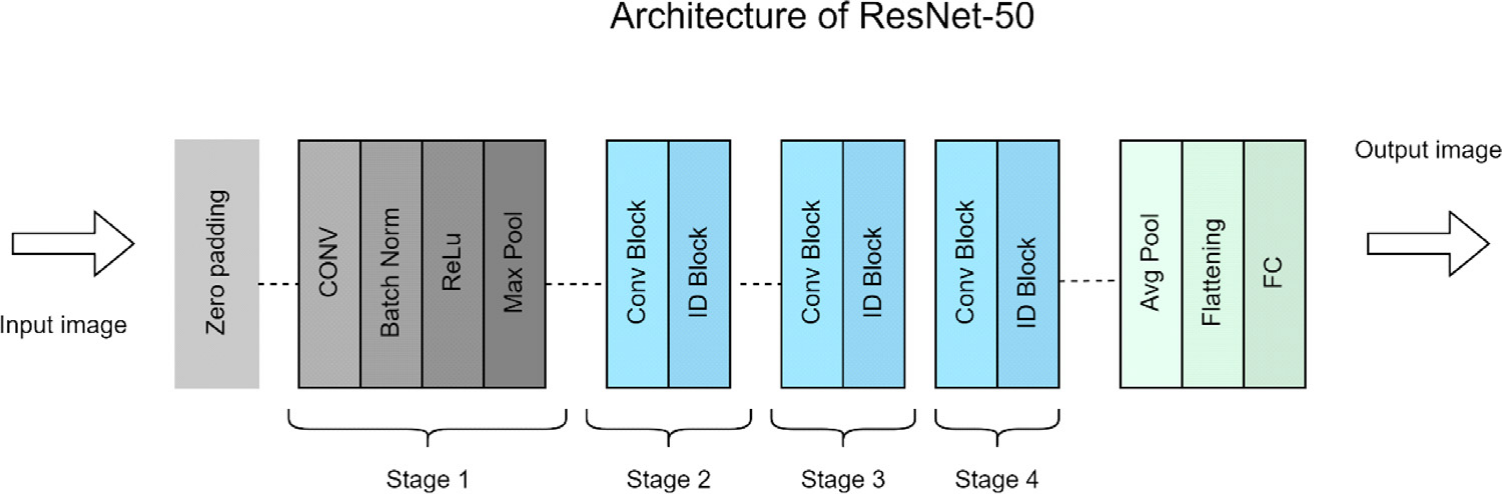
Architecture of REsNet-50.

After applying augmentation in our real dataset, we divided our dataset into three parts: train, test, and validation. The percentage of division are 70 %, 15 % and 15 % respectively. The images are selected randomly for those parts. Fig. 2 gives us a clear idea of our work and represents the classification process. In the data preprocessing part, we use three techniques: rescaling, augmentation, and noise reduction. By using the training dataset, we train resNet50 model for our work. Then we use validation dataset to evaluate the model performance and tune the model hyperparameters. The validation part helps models to identify unseen data. And finally, we use test dataset to test our model. The test part helps us to identify how well the model performs to new or unseen data (Fig. 3).

ResNet-50 form with 48 convolution layers,1 max pooling and 1 average pool layer [8], that structured into residual blocks, which has feature like shortcut connections, approve for gradients to flow direction by using the network layers. In figure-3 we see that, the architecture of ResNet-50 begins with a convolutional layer and a max-pooling layer to identify basic features and minimize the spatial size of input images. In the training part of ResNet-50 on our dataset, we set the training data for ResNet-50 model as a input image. Then it proceeds into residual blocks with four stages. Each stage works for increasing depth and filter sizes by using multiple convolutional layers. By using convolutional shortcuts, these blocks create direct paths for gradient and simplify more effective backpropagation. The final layers, based on global average pooling and fully connected layer gives classification output. In this training process also used normalization and ReLU activation function for accelerate the training process. In the training part we also keep records of training accuracy and loss for a better understanding of model learning. After completing training process, we set our testing data for evaluating the model. Also, we keep records for validation same as training. Then we just plotting that recorded data and find the differences of training and validation part. I we clearly understand how well the model is learning and classify the unknown data.

**Fig. 4:**
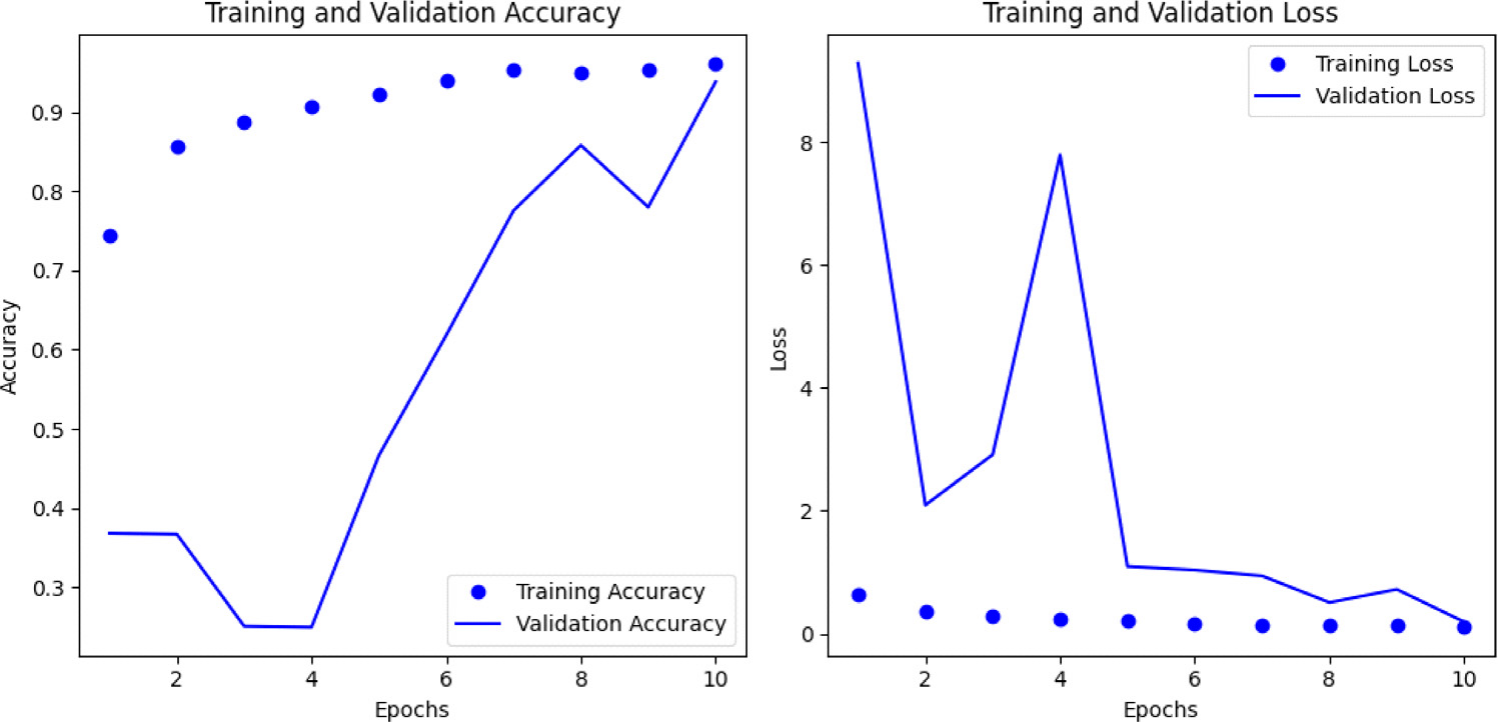
Performance of ResNet50 model.

In Fig. 4, we see that model training part accuracy gradually increased. During the training phase, the maximum accuracy of 96 % was attained at 10 number epochs. However, we don't see such kind of case in the validation section. First few epochs decrease and then increase and finally find nearly train accuracy. Also, we see that train loss in natural but validation loss first 5 epochs are not natural but after that it become like train loss. Following that procedure, we test our model and discover that it has a respectable 94 % accuracy. Fig. 4 demonstrates that (Table 4).

**Table 4:** Test accuracy of ResNet50 model.

Classes of the dataset	Model name	Test accuracy
Anthracnose leaf spot, Healthy, Straw mite	ResNet50	0.94

In the future, we intend to combine machine learning methods and artificial intelligence for image processing to develop a mobile application that assists users in identifying diseases in Malabar spinach.

## Limitations

There are some limitations:

• Low amount of data
• Only work with Malabar spinach leaf

## Ethics Statement

The authors of this article did not conduct any studies with humans or animals as subjects. Datasets included in this paper are publicly available, though it is important to cite sources correctly.

## CRediT Author Statement

Mushfiqur Rahman: Dataset Curation, Data Collection, Data Labeling, Dataset Publication, Supervision, Writing– review & editing. Md Al Mamun: Writing – original draft, Methodology.

## Data Availability

Mendeley DataMalabar Spinach dataset for diseases classification using deep learning approach (Original data)
